# The Role of Unfinished Business in the Relationship Between Attachment Style and Grief: A Two-Wave Longitudinal Study

**DOI:** 10.3390/bs16030355

**Published:** 2026-03-02

**Authors:** Suqin Tang, Jingjing Huang, Zifeng Yang

**Affiliations:** 1 School of Psychology, Shenzhen University, Shenzhen 518060, China; zfyang2026@163.com; 2 The Shenzhen Humanities and Social Sciences Key Research Bases of the Center for Mental Health, Shenzhen University, Shenzhen 518060, China; 3 Key Laboratory of Applied Experimental Psychology, National Demonstration Center for Experimental Psychology Education, Faculty of Psychology, Beijing Normal University, Beijing 100875, China

**Keywords:** grief, attachment, unfinished business, longitudinal study, mediation

## Abstract

Grounded in attachment theory, the present study aimed to examine the predictive effects of deceased-specific attachment styles (anxious and avoidant) on grief and to test the mediating roles of two dimensions of unfinished business, namely unfulfilled wishes and unresolved conflicts. Using a two-wave longitudinal design, we conducted a 3-month follow-up survey (T1: October 2024; T2: January 2025) among Chinese adults who experienced bereavement within the past five years. Participants completed the Experiences in Close Relationships—Relationship Structures questionnaire at T1 and the Unfinished Business in Bereavement Scale and the International Prolonged Grief Disorder Scale at both T1 and T2. A total of 206 participants (Mage = 25.07 years; 51.94% women) completed both assessments. After controlling for T1 grief, demographic- and loss-related covariates, T1 deceased-specific attachment anxiety and attachment avoidance did not directly predict T2 grief. However, T1 deceased-specific attachment avoidance significantly negatively predicted T2 unfulfilled wishes, and both unfulfilled wishes and unresolved conflicts at T2 significantly positively predicted T2 grief. Furthermore, T2 unfulfilled wishes significantly mediated the relationship between T1 deceased-specific attachment avoidance and T2 grief. Thus, directly targeting unfinished business may serve as a more efficient and specific clinical strategy than broad attachment-focused interventions.

## 1. Introduction

Losing a loved one is among the most profound and universal experiences in human life. Regardless of cultural background or life stage, bereavement often disrupts an individual’s established life order and triggers intense emotional distress and adjustment challenges. In response to loss, people commonly experience grief that manifests across physiological, emotional, cognitive, and behavioral domains (M. K. Shear, 2015). For some bereaved individuals, grief may persist and intensify over time, developing into Prolonged Grief Disorder (PGD) (American Psychiatric Association, 2022; Prigerson et al., 2021; World Health Organization, 2018). A recent meta-analysis of bereaved Chinese individuals reported a pooled prevalence of 32.4% (95% CI: 18.2–50.8%) for grief and 8.9% (95% CI: 4.2–17.6%) for PGD (Yuan et al., 2024), underscoring the widespread and serious nature of this issue.

To systematically understand PGD, researchers have proposed several theoretical frameworks and have sought to identify its risk factors and underlying mechanisms. Among these, K. Shear and Shair’s (2005) attachment-based model suggests that, during the long-term adaptation process, the bereaved person’s attachment style may shape their coping strategies, leading to different grief outcomes. Therefore, exploring the relationship between attachment style and grief, as well as clarifying the psychological mechanisms that may mediate this link, is of great clinical significance for developing targeted intervention strategies that alleviate psychological distress among the bereaved and reduce the incidence of PGD. Specifically, the present study adopts a longitudinal design to determine whether deceased-specific attachment styles are associated with subsequent grief and whether unfinished business (i.e., unfulfilled wishes and unresolved conflicts) may mediate this association.

### 1.1. Attachment Style and Grief

In human relationships, attachment is a universal and profound phenomenon that reflects the innate tendency to form emotional bonds with significant others, providing a sense of safety and comfort (Bowlby, 1969). Initially, it manifests in the attachment between infants and their primary caregivers, and as individuals grow, their attachment figures gradually extend to other important people in their lives (Bowlby, 1973; Weiss, 2006). Accordingly, many theories of loss and grief have emphasized the crucial role of attachment (e.g., Maccallum & Bryant, 2013; K. Shear & Shair, 2005).

It is noteworthy that different attachment styles may lead to distinct adjustment processes following loss. The prevailing view conceptualizes attachment in terms of two core dimensions: attachment anxiety and avoidance (Fraley & Shaver, 2000; Fraley & Spieker, 2003). Early studies have suggested that insecure attachment, characterized by high anxiety or avoidance, serves as an important risk factor for grief (Bowlby, 1980; Maccallum & Bryant, 2013; K. Shear & Shair, 2005).

Although both attachment anxiety and avoidance have been identified as risk factors, researchers generally agree that they may influence post-loss adaptation in different ways. Attachment anxiety reflects the degree to which individuals worry about being rejected or abandoned by their attachment figures, or about the unavailability of support when needed (Fraley & Shaver, 2000; Mikulincer et al., 2002). Individuals high in attachment anxiety tend to continue “searching” for the deceased after the loss, constantly seeking confirmation of the deceased’s availability and presence. However, the reality of the loss repeatedly activates their attachment system, which may lead to prolonged and maladaptive grief (Bowlby, 1980; Shaver & Fraley, 2008).

In contrast, attachment avoidance reflects the tendency to maintain emotional distance from attachment figures and to value independence and self-reliance (Fraley & Shaver, 2000; Mikulincer et al., 2002). Individuals high in attachment avoidance often suppress emotional expression and avoid painful thoughts and feelings related to the loss, which may hinder the integration of the loss into autobiographical memory. As a result, even mild reminders of the deceased can later trigger persistent and maladaptive grief due to insufficient emotional adaptation (Bowlby, 1980; Shaver & Fraley, 2008).

In cross-sectional studies, most evidence supports that general attachment anxiety and avoidance are both positively associated with grief (e.g., Captari et al., 2021; Gegieckaite & Kazlauskas, 2020; Sekowski & Prigerson, 2022), although a few studies have reported nonsignificant associations (Burke et al., 2019; Levi-Belz & Lev-Ari, 2019). Furthermore, after controlling for demographic and loss-related variables, both attachment anxiety and avoidance remain positively associated with concurrent grief (Boelen & van den Bout, 2010; Meier et al., 2013; Sekowski & Prigerson, 2022; Yu et al., 2016). However, some studies have found a negative association between attachment avoidance and concurrent grief (LeRoy et al., 2020). In longitudinal studies, after controlling for baseline grief, neither dimension significantly predicts subsequent grief (Suttle et al., 2022), nor are they associated with the improvement of grief from T1 to T2 (Meert et al., 2011).

Because individuals’ attachment styles can vary across different interpersonal relationships (Fraley et al., 2011; Smigelsky et al., 2020), some studies have examined deceased-specific attachment styles, suggesting that they may predict grief more effectively than general attachment styles (Jerga et al., 2011). However, the findings remain inconsistent. Regarding deceased-specific attachment anxiety, most studies have found it to be positively correlated with grief (Black et al., 2019; Delespaux et al., 2013; Jerga et al., 2011; Smigelsky et al., 2020), though some have reported no significant correlation (Huh et al., 2017). For deceased-specific attachment avoidance, several studies have reported a positive correlation with grief (Black et al., 2019; Huh et al., 2017), while others have found no significant correlation (Jerga et al., 2011), or even a negative correlation (Delespaux et al., 2013; Smigelsky et al., 2020).

A recent meta-analysis provided integrative evidence addressing the above inconsistencies, showing that both attachment anxiety and avoidance were positively correlated with grief, though with small effect sizes (anxiety: r = 0.28, 95% CI [0.23, 0.32]; avoidance: r = 0.21, 95% CI [0.16, 0.25]). Further analyses revealed no significant difference between general and deceased-specific attachment anxiety in their correlations with grief, whereas the correlation for general attachment avoidance was significantly stronger than that for deceased-specific attachment avoidance (Eisma et al., 2023). Despite these findings, more research is needed to further examine how attachment styles, particularly deceased-specific attachment styles, affect grief. Therefore, the present study aims to examine how deceased-specific attachment anxiety and attachment avoidance are associated with grief.

### 1.2. The Mediating Role of Unfinished Business

Although attachment style provides a crucial perspective for understanding grief, its specific underlying mechanisms remain to be uncovered. Over the past decades, there has been growing research interest in identifying potential mediating mechanisms linking insecure attachment to psychological distress (Remondi et al., 2023). In the context of loss, because attachment style reflects the emotional bond between the bereaved individual and the deceased, difficulties in this continuing attachment, manifested as unfinished business, may represent a potential mechanism through which attachment influences grief (Holland et al., 2020).

Unfinished business (UB) refers to the incomplete, unexpressed, or unresolved relational issues between the bereaved individual and the deceased that cause emotional distress (Holland et al., 2014; Huang et al., 2025). This phenomenon is quite common among the bereaved, with over 40% reporting the presence of unfinished business with the deceased (Klingspon et al., 2015). Researchers have further divided unfinished business into two heterogeneous dimensions: unfulfilled wishes (UWs) and unresolved conflicts (UCs) (Holland et al., 2020). The former refers to unrealized shared goals, unfulfilled promises, or unexpressed positive emotions with the deceased, whereas the latter involves unresolved arguments, unaddressed secrets, or internal conflicts.

#### 1.2.1. Unfinished Business and Attachment Style

Unfinished business can be understood as a form of maladaptive emotional memory that gradually develops through a series of interactions with significant others (Greenberg & Paivio, 1997). Therefore, as the core template for relational interactions, attachment may exert a systematic influence on the formation and development of unfinished business. Specifically, individuals with different attachment styles show distinct patterns in relationship building, emotional expression, and conflict resolution, which in turn shape both the likelihood and the characteristics of their unfinished business.

For individuals high in attachment anxiety, they constantly seek closeness and reassurance from their attachment figures but often still feel that their needs are not adequately met (Wei et al., 2005). They tend to adopt hyperactivating strategies, displaying heightened emotionality in their efforts to gain the attachment figure’s attention (Mikulincer & Shaver, 2003, 2016). Such behavior may hinder constructive interactions with attachment figures (Li & Chan, 2012), thereby increasing the likelihood of accumulating unfinished business in the relationship. After the loss, they may also ruminate more about these unresolved issues and experience stronger distressing emotions such as guilt and shame (Gross & Hansen, 2000; Leonardi et al., 2022).

In contrast, individuals high in attachment avoidance tend to maintain emotional distance from their attachment figures and avoid showing excessive dependence or needs. They adopt deactivating strategies, often suppressing the expression of intimacy and affection, and may reject or disregard support from their attachment figures (Mikulincer & Shaver, 2003, 2016). As a result, they may be less likely to perceive or acknowledge the presence of unfinished business in their relationships. After the loss, they may also tend to minimize or deny the existence of unfinished business and related emotions, thereby experiencing relatively less emotional distress.

To date, only one study has preliminarily examined the relationship between unfinished business and attachment style, finding that unfinished business and its two dimensions were positively correlated with attachment anxiety and negatively correlated with attachment avoidance. Moreover, the correlation between unresolved conflicts and attachment anxiety was stronger, whereas the correlation between unfulfilled wishes and attachment avoidance was stronger (Holland et al., 2020). These findings offer preliminary evidence for understanding the differentiated associations between two attachment styles and two dimensions of unfinished business. Therefore, the present study aims to examine whether deceased-specific attachment anxiety and attachment avoidance show distinct associations with unfulfilled wishes and unresolved conflicts.

#### 1.2.2. Unfinished Business and Grief

From the perspective of Gestalt therapy, emotions are closely tied to individual needs, serving to guide action toward need fulfillment and the achievement of closure. When emotions are suppressed, unmet needs may turn into unfinished business (Paivio, 1994). Such unfinished business continues to occupy cognitive resources, hinders open perception of new situations, and is easily reactivated by similar contexts (Greenberg & Safran, 1987; Perls et al., 1951). This not only obstructs individuals from modifying problematic interaction patterns from the past but also limits their ability to learn new adaptive ones, thereby impairing overall psychological functioning and development (Perls et al., 1951). For the bereaved, if a large amount of unfinished business has accumulated in their long-term relationship with the deceased, the loss event may reactivate these memories, causing them to remain preoccupied with the past and struggle to adapt to life without the deceased (Malcolm, 1999; Tobin, 1971).

Consistent with theoretical accounts, unfinished business and its two dimensions have been consistently associated with greater grief (Holland et al., 2020; Klingspon et al., 2015; Lee et al., 2022). In particular, unfinished business remained positively associated with concurrent grief even after controlling for meaning made of the loss, attachment style, relationship quality, family income, and relationship to the deceased (Holland et al., 2020). However, when demographic- and loss-related variables and specific emotions (regret, shame, and guilt) were controlled, only unresolved conflicts showed a unique association with grief, whereas unfulfilled wishes did not (Lee et al., 2022). Accordingly, the present study aims to examine whether unfulfilled wishes and unresolved conflicts play distinct roles in grief recovery.

### 1.3. The Current Study

In summary, although previous studies have examined the effects of attachment style on grief, the findings remain inconsistent, with particular controversy surrounding the role of attachment avoidance. Moreover, attachment style may vary across different relationships, and research focusing on the relationship between deceased-specific attachment style and grief is still limited, especially longitudinal studies capable of testing the predictive effects of the former on the latter (Eisma et al., 2023). In addition, the underlying mechanisms through which deceased-specific attachment style influences grief have not been sufficiently explored. Theoretically, unfinished business may serve as an important mediator, and its two distinct dimensions (i.e., unfulfilled wishes and unresolved conflicts) may play different roles.

Based on this rationale, the present study adopts a longitudinal design to examine whether and how deceased-specific attachment styles influence grief through different dimensions of unfinished business (unfulfilled wishes and unresolved conflicts). We hypothesize that: (1) after controlling for baseline grief, deceased-specific attachment anxiety would positively predict subsequent grief, whereas deceased-specific attachment avoidance would negatively predict subsequent grief; and (2) unfinished business would mediate the relationship between deceased-specific attachment style and grief, with mediating effects that differ across dimensions. Specifically, attachment anxiety would show a stronger association with unresolved conflicts, whereas attachment avoidance would show a stronger association with unfulfilled wishes, thereby exerting differentiated influences on grief.

## 2. Methods

### 2.1. Procedures

The study protocol was reviewed and approved by the Ethics Committee of the first author’s affiliated institution (SZUPSY2023019). The present study adopted a combination of convenience sampling and snowball sampling to recruit voluntary adult participants who had experienced loss. Recruitment materials containing a QR code linked to the survey were distributed via multiple online social platforms, including WeChat groups and Moments. Data collection was conducted through the Wenjuanxing online platform (https://www.wjx.cn (accessed on 31 January 2025)).

In the T1 survey (October 2024), an informed consent form was presented on the first page of the questionnaire, detailing the purpose and significance of the study, the confidentiality of data, and participants’ right to withdraw at any time. Participants then completed a series of questionnaires, followed by the provision of contact information at the end of the survey. Three months later, participants who met the inclusion criteria at T1 were contacted via WeChat and invited to complete the T2 (January 2025) questionnaire, which served as the second wave of data collection. Key loss-related information (e.g., time since loss, relationship to the deceased) was cross-checked across the two waves to ensure consistency. Participants who completed each survey in full and met the validity criteria received monetary compensation.

### 2.2. Participants

The inclusion criteria for bereaved participants in the present study were as follows: (1) aged 18 years or older; (2) having experienced the death of a significant other (screened using a single item, “How important was the deceased to you?” rated on a 5-point Likert scale, with 1 = “not important” and 5 = “very important”; only participants who rated 4 or 5 were included); (3) the loss occurred within the past five years (i.e., after September 2019); and (4) correct response to the lie-detection item. The first survey (T1) was conducted in October 2024, and a total of 405 participants met the inclusion criteria. Some participants were lost to follow-up due to being unreachable via WeChat. The second survey (T2) was conducted in January 2025, yielding 283 returned questionnaires. Consistency checks were then performed on key loss-related information across the two surveys (e.g., time since loss, relationship to the deceased), and 77 participants were excluded for inconsistency. Consequently, the final valid sample included 328 participants at T1 (100%) and 206 participants at T2 (62.8%). The 206 participants who completed both surveys constituted the final analytic sample of the present study.

### 2.3. Measures

#### 2.3.1. Basic Information

Basic information included: (1) demographic variables, namely age, gender, and educational level; (2) loss-related variables, including the age and gender of the deceased, time since loss (in months), relationship to the deceased (e.g., parent, grandparent), cause of death (e.g., illness, accident), and the perceived importance of the deceased.

#### 2.3.2. Experiences in Close Relationships—Relationship Structures Scale (ECR-RS)

The Experiences in Close Relationships—Relationship Structures Scale (ECR-RS; Fraley et al., 2011) was used to assess participants’ attachment style within their relationship with the deceased. The scale consists of nine items covering two dimensions: attachment anxiety (3 items; e.g., “I worry that he/she doesn’t care about me as much as I care about him/her”) and attachment avoidance (6 items; e.g., “I prefer not to show him/her how I feel deep down”). Participants rated each statement on a 7-point Likert scale (1 = “strongly disagree,” 7 = “strongly agree”). Mean scores for each dimension were computed, with higher scores indicating a stronger tendency toward that attachment dimension. In the present study, Cronbach’s α was 0.817 for the overall ECR-RS, 0.930 for attachment anxiety, and 0.819 for attachment avoidance.

#### 2.3.3. Unfinished Business in Bereavement Scale (UBBS)

The Unfinished Business in Bereavement Scale (UBBS; Holland et al., 2020) was used to assess participants’ unfinished business with the deceased. The scale consists of 28 items and includes two dimensions: unfulfilled wishes (16 items; e.g., “I wish I had told him/her how important he/she was to me”) and unresolved conflicts (12 items; e.g., “There were important issues or conflicts in our relationship that I never resolved”). Participants rated the degree of distress they felt over the past month for each statement on a 5-point Likert scale (1 = “not at all distressed,” 5 = “extremely distressed”). Mean scores were computed for each dimension, with higher scores indicating greater subjective distress within that domain. The Chinese version of the UBBS has demonstrated good reliability and validity among Chinese bereaved adults (Huang et al., 2024). In the present study, Cronbach’s α was 0.922 for T1 UBBS, 0.914 for T1 unfulfilled wishes, and 0.900 for T1 unresolved conflicts; and 0.948 for T2 UBBS, 0.940 for T2 unfulfilled wishes, and 0.925 for T2 unresolved conflicts.

#### 2.3.4. International Prolonged Grief Disorder Scale (IPGDS)

The International Prolonged Grief Disorder Scale (IPGDS; Killikelly et al., 2020), which was developed based on the World Health Organization’s International Classification of Diseases, 11th Revision (ICD-11) diagnostic criteria for PGD, was used to assess participants’ grief severity. The present study only administered the 13-item standard scale, which covers separation distress (items 1–2), associated emotional distress (items 3–12), and functional impairment (item 13). Participants rated each item according to how often they experienced these feelings during the past month, using a 5-point Likert scale (1 = “almost never,” 5 = “always”). The mean score across items 1–13 was computed to index grief severity, with higher scores indicating greater grief. In the present study, Cronbach’s α was 0.925 for T1 IPGDS and 0.937 for T2 IPGDS.

### 2.4. Data Analysis

Data analyses were conducted using SPSS 28.0 and Mplus 8.3. First, descriptive statistics and correlation analyses were performed for demographic and loss-related variables, deceased-specific attachment styles, unfinished business, and grief. For categorical variables, independent-sample *t*-tests and chi-square tests were used to examine group differences in grief severity, and variables showing significant differences were included as covariates in subsequent structural equation modeling. Third, after controlling for T1 grief, T1 deceased-specific attachment anxiety and T1 deceased-specific attachment avoidance were entered as independent variables, with T2 grief as the dependent variable, to examine direct effects. Finally, a mediation model was constructed, in which T1 deceased-specific attachment anxiety and T1 deceased-specific attachment avoidance served as independent variables, T2 unfulfilled wishes and T2 unresolved conflicts (controlling for their T1 levels) served as mediators, and T2 grief (controlling for T1 grief) served as the dependent variable. The mediating effects of unfulfilled wishes and unresolved conflicts were tested using a nonparametric percentile bootstrap method with 5000 resamples.

## 3. Results

### 3.1. Sample Characteristics

Among the 206 participants included in the final analysis, 99 were men (48.06%) and 107 were women (51.94%), with ages ranging from 18 to 67 years (M = 25.07, SD = 6.41). The duration of loss ranged from 3 to 44 months (M = 16.19, SD = 9.79). Additional demographic and loss-related information for the final sample and the attrition sample is presented in Table 1.

Participants who completed both surveys and those who completed only the first survey did not differ significantly in demographic or loss-related variables, nor in T1 deceased-specific attachment avoidance (t(326) = 0.66, *p* = 0.51), T1 deceased-specific attachment anxiety (t(326) = −0.58, *p* = 0.56), or T1 unfulfilled wishes (t(326) = −1.92, *p* = 0.06). However, significant differences emerged in T1 unresolved conflicts (t(326) = 4.27, *p* < 0.001) and in T1 grief (t(326) = 2.84, *p* = 0.005). Participants in the attrition sample showed higher levels of T1 unresolved conflicts and more severe T1 grief.

### 3.2. Correlations Among Attachment Style, Unfinished Business, and Grief

Descriptive statistics and correlations for the variables are shown in Table 2. T1 deceased-specific attachment anxiety showed a significant positive correlation with T1 unfulfilled wishes (r = 0.23, *p* = 0.001) but no significant correlation with T2 unfulfilled wishes. It was positively correlated with both T1 and T2 unresolved conflicts (rT1 = 0.41, *p* < 0.001; rT2 = 0.32, *p* < 0.001) and with T1 and T2 grief (rT1 = 0.36, *p* < 0.001; rT2 = 0.29, *p* < 0.001). T1 deceased-specific attachment avoidance was negatively correlated with T1 and T2 unfulfilled wishes (rT1 = −0.29, *p* < 0.001; rT2 = −0.30, *p* < 0.001) and with T1 and T2 grief (rT1 = −0.28, *p* < 0.001; r = −0.17, *p* = 0.016), but showed no significant correlations with T1 or T2 unresolved conflicts. Both T1 and T2 unfulfilled wishes (r = 0.40–0.57, *p*s < 0.001) and T1 and T2 unresolved conflicts (r = 0.47–0.70, *p*s < 0.001) were positively correlated with T1 and T2 grief.

### 3.3. Predictive Effects of Specific Attachment Styles on Grief

Analyses examining the associations between demographic and loss-related variables and grief revealed that the age of the deceased was negatively correlated with both T1 and T2 grief (r = −0.19, *p* = 0.007; r = −0.22, *p* = 0.001). A significant difference was found in T2 grief between participants who lost men (M = 2.65) and those who lost women (M = 2.95; *p* = 0.017). In addition, participants who experienced a loss due to an accident (M = 3.55) reported significantly higher T1 grief than those whose loss was due to a chronic illness (M = 3.04; *p* = 0.008). Therefore, age of the deceased, gender of the deceased, and cause of death were included as covariates in the subsequent analyses.

**Figure 1:**
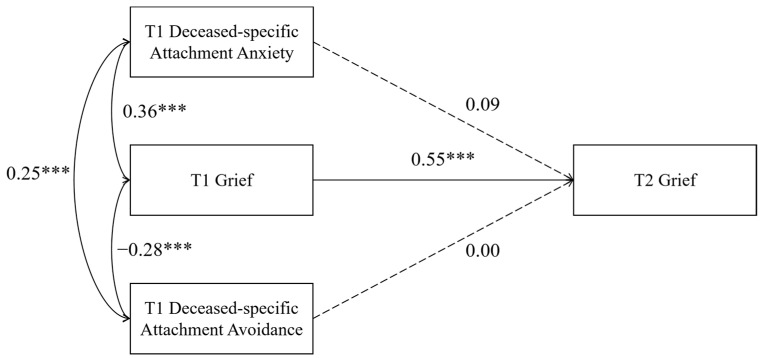
Direct effect model. Covariates (deceased’s age, gender, and cause of death) are omitted for clarity. Note. *** *p* < 0.001.

The results of the direct effect analysis indicated that the model was saturated, with the following fit indices: χ^2^/df = 0.000, RMSEA = 0.000, CFI = 1.000, TLI = 1.000, and SRMR = 0.000. As shown in Figure 1, after controlling for T1 grief, neither T1 deceased-specific attachment anxiety nor T1 deceased-specific attachment avoidance significantly predicted T2 grief (β = 0.09, *p* > 0.05; β = 0.00, *p* > 0.05).

### 3.4. Mediating Effects of Unfinished Business in the Relationship Between Specific Attachment Styles and Grief

**Figure 2:**
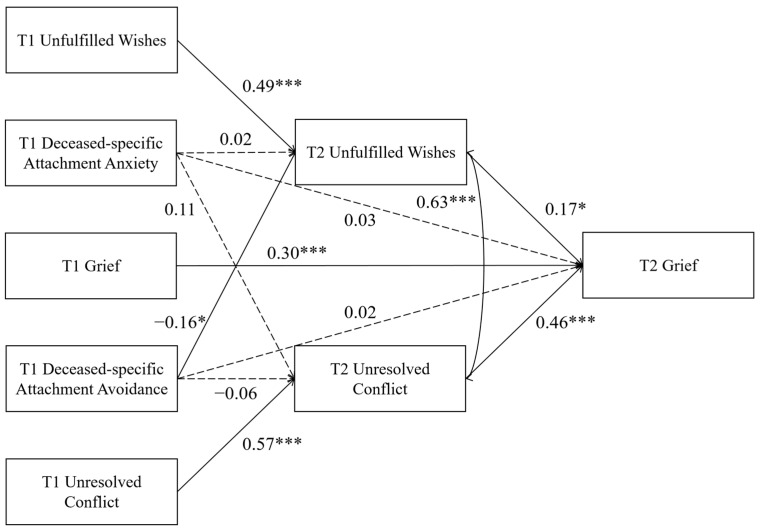
The mediation model. Correlations among T1 variables and covariates (deceased’s age, gender, and cause of death) are omitted for clarity. Note. * *p* < 0.05, *** *p* < 0.001.

The mediation model showed a good fit to the data: χ^2^/df = 1.77, RMSEA = 0.061, CFI = 0.980, TLI = 0.956, and SRMR = 0.029. As shown in Figure 2, after controlling for the baseline level of each outcome variable, T1 deceased-specific attachment anxiety did not significantly predict T2 unfulfilled wishes (β = 0.02, *p* > 0.05) or T2 unresolved conflicts (β = 0.11, *p* > 0.05). T1 deceased-specific attachment avoidance remained a significant negative predictor of T2 unfulfilled wishes (β = −0.16, *p* = 0.041) but did not significantly predict T2 unresolved conflicts (β = −0.06, *p* > 0.05). When controlling for T1 grief, neither T1 deceased-specific attachment anxiety (β = 0.03, *p* > 0.05) nor T1 deceased-specific attachment avoidance (β = 0.02, *p* > 0.05) significantly predicted T2 grief. However, both T2 unfulfilled wishes and T2 unresolved conflicts were significant positive predictors of T2 grief (β = 0.17, *p* = 0.033; β = 0.46, *p* < 0.001).

The mediation analysis further revealed that the 95% confidence interval for the indirect path T1 deceased-specific attachment avoidance → T2 unfulfilled wishes → T2 grief did not include 0 (95% CI = [−0.082, −0.003]), indicating that T2 unfulfilled wishes significantly mediated the relationship between T1 deceased-specific attachment avoidance and T2 grief.

## 4. Discussion

The present study adopted a two-wave longitudinal design to examine the impact of deceased-specific attachment style on grief. For the first time, it constructed a path model in which the two dimensions of unfinished business (unfulfilled wishes and unresolved conflicts) served as mediators. The results indicated that neither deceased-specific attachment anxiety nor attachment avoidance directly predicted grief severity three months later. However, deceased-specific attachment avoidance indirectly influenced grief through unfulfilled wishes. In addition, unresolved conflicts were found to positively predict grief. These findings provide longitudinal evidence for the relationship between specific attachment styles and grief, offering new insights into the mechanisms through which attachment styles influence adaptation to loss.

### 4.1. The Relationship Between Attachment Style and Grief

Deceased-specific attachment anxiety was positively associated with concurrent grief, a finding consistent with most previous studies (Eisma et al., 2023). Individuals with higher levels of attachment anxiety toward the deceased often repeatedly recall shared experiences and overly focus on cues related to the deceased, attempting to re-experience the closeness and security once provided. This tendency, in turn, leads to higher levels of grief (Shaver & Fraley, 2008; M. Stroebe et al., 2010). However, deceased-specific attachment avoidance was negatively associated with concurrent grief, which contradicts the results of the meta-analysis (Eisma et al., 2023) but aligns with negative associations observed in some Western studies (Delespaux et al., 2013; Smigelsky et al., 2020). Importantly, our findings extend this negative concurrent association to a Chinese cultural context, complementing prior Chinese work that has focused on general attachment avoidance (Yu et al., 2016). From a theoretical perspective, higher attachment avoidance toward the deceased is characterized by deactivating the attachment system, which may reduce attachment-related thoughts and emotions, dampen engagement with loss-related cues, and foster self-reliance and daily functioning. This reduced engagement with grief-related cues likely leads to lower levels of concurrent grief (Fraley & Bonanno, 2004; Smigelsky et al., 2020).

Notably, such discrepancies may also, to some extent, be attributable to differences in measurement instruments and assessment approaches across studies. Both the present study and Smigelsky et al. (2020) assessed attachment avoidance toward the deceased using the ECR-RS, whose wording allows it to capture various relational contexts (e.g., parents, partners, friends) and thus makes it particularly suitable for evaluating attachment to the deceased, independent of relationship type. By contrast, although the ECR-R was designed to assess general attachment style, it may in practice primarily reflect attachment patterns in romantic relationships (Fraley et al., 2011). Furthermore, because attachment levels toward the deceased differ significantly from general attachment levels (Smigelsky et al., 2020), the present study explicitly specified the target relationship as the deceased, which likely enhanced measurement precision and specificity. Given this, future studies should explore the relationship between deceased-specific attachment avoidance and grief in greater detail.

However, after controlling for baseline grief, neither deceased-specific attachment anxiety nor attachment avoidance significantly predicted future grief. While previous longitudinal studies have largely focused on the impact of general attachment styles on grief, the present study suggests that deceased-specific insecure attachment may not play a significant role in long-term grief. Research has shown that attachment functions are part of a hierarchical network, and these functions can transfer from one attachment figure to another after major life events (Gillath et al., 2019). Accordingly, one possible explanation is that, over time, individuals’ everyday life demands prompt them to reinvest emotional energy into other meaningful relationships. Surviving attachment figures may partially substitute the role of the deceased, which can diminish the psychological impact of the prior attachment bond (LeRoy et al., 2020; Mikulincer & Shaver, 2022). This perspective is also supported by Maciejewski et al. (2022), who proposed that the loss of a loved one creates a vacancy in an individual’s social network, which must be filled by strengthening existing relationships or establishing new ones.

Moreover, this substitution process may be more readily facilitated within the Chinese cultural context, which is often characterized by Confucian-influenced collectivism (Agishtein & Brumbaugh, 2013). Following bereavement, family responsibilities and role obligations may prompt individuals to redirect emotional investment toward surviving relationships. As a result, deceased-specific attachment may be less likely to have significant prospective effects beyond baseline grief. Overall, attachment relationships seem to change dynamically as time passes and as new connections are formed (Fraley et al., 2011, 2021). Thus, deceased-specific attachment style may be better viewed as a time-sensitive indicator of concurrent grief rather than a fixed, time-invariant vulnerability factor for future grief.

### 4.2. The Mediating Role of Unfinished Business

Consistent with previous studies, the present research found that both unfulfilled wishes and unresolved conflicts were significantly and positively correlated with grief (Lee et al., 2022). More importantly, after controlling for baseline grief, both T2 unfulfilled wishes and T2 unresolved conflicts continued to positively predict T2 grief, extending the conclusions of prior longitudinal research. Previous evidence has shown that unfinished business, even after controlling for baseline grief, consistently predicts higher grief levels three months later, indicating its role as a prospective risk factor for grief (Huang et al., 2025). The current findings additionally reveal that the persistent presence of unfulfilled wishes and unresolved conflicts plays an immediate and maintaining role in sustaining bereaved individuals’ concurrent grief. Thus, these two dimensions of unfinished business can be considered proximal risk factors for grief. Notably, although unfinished business itself is a risk factor, whether it exacerbates the severity of grief may depend on how the bereaved individual addresses it (i.e., whether through continuing bonds or other coping strategies, such as avoidance). Adaptive coping strategies may buffer grief, while maladaptive strategies could intensify grief responses and potentially lead to PGD.

Building on these findings, the mediation analysis revealed that T2 unfulfilled wishes mediated the relationship between T1 deceased-specific attachment avoidance and T2 grief. Previous research has shown that deceased-specific attachment avoidance is significantly and negatively correlated with unfulfilled wishes (Holland et al., 2020). The present study further demonstrated that, even after controlling for T1 unfulfilled wishes, T1 deceased-specific attachment avoidance remained a significant negative predictor of T2 unfulfilled wishes. Individuals high in attachment avoidance tend to suppress their emotional needs and dependence within close relationships (Fraley & Shaver, 2000). After bereavement, they may consciously or unconsciously minimize their focus on unfulfilled wishes related to the deceased as a self-protective strategy to avoid emotional distress, which leads to lower levels of distress regarding unfulfilled wishes. This emotional avoidance strategy may have a lasting effect, alleviating not only immediate distress about unfulfilled wishes but also reducing future distress over them. Such avoidance of emotional distress, in turn, lessen emotional involvement and rumination during bereavement adaptation, thereby contributing to lower levels of grief.

However, T2 unresolved conflicts did not mediate the relationship between T1 deceased-specific attachment avoidance and T2 grief. The primary reason for this is that attachment avoidance failed to significantly predict subsequent levels of unresolved conflicts. Unlike previous findings (Holland et al., 2020), the present study found no significant correlation between deceased-specific attachment avoidance and unresolved conflicts, nor any longitudinal predictive effect. One possible explanation is that, within the Chinese cultural context, which emphasizes restrained expression and interpersonal harmony (Markus & Kitayama, 1991), individuals high in attachment avoidance may be more likely to adopt culturally shaped cognitive strategies that “set aside” conflict-related memories with the deceased or “let bygones be bygones” (e.g., “the person has passed away, so there is no need to dwell on it”). Meanwhile, cross-cultural evidence indicates that bereaved individuals in the more collectivistic Chinese context report expressing conflict-related emotions (e.g., anger and blame/guilt) less than they believe they should, and less than bereaved individuals in a more individualistic Swiss German context (Zhou et al., 2023). Under such display rules, conflict-related appraisals may be less consistently expressed or processed overtly, potentially weakening the observable association between attachment avoidance and unresolved conflicts. However, such cognitive regulation strategies may not truly reduce the implicit, underlying sense of unresolved conflict.

In addition, it is noteworthy that T2 unfulfilled wishes and T2 unresolved conflicts did not mediate the relationship between T1 deceased-specific attachment anxiety and T2 grief. This was mainly because T1 attachment anxiety did not significantly predict either T2 unfulfilled wishes or T2 unresolved conflicts. Although deceased-specific attachment anxiety was positively correlated with concurrent unfulfilled wishes and unresolved conflicts (Holland et al., 2020), suggesting that individuals with higher attachment anxiety may be more prone to deliberately ruminate on loss-related scenes and emotions (Huh et al., 2020), repeatedly reflecting on their unfulfilled wishes and unresolved conflicts with the deceased and thereby experiencing stronger immediate distress, its influence may be overshadowed by other grief processes that evolve over time. Consequently, deceased-specific attachment anxiety did not exhibit a sustained reinforcing effect on subsequent unfinished business.

In summary, integrating the Integrative Risk Factor Framework for the Prediction of Bereavement Outcome (M. S. Stroebe et al., 2006) and based on the present findings, the present study suggests that the reason why existing research has not consistently demonstrated a prospective effect of deceased-specific attachment styles on grief (Eisma et al., 2023) may be that attachment style functions as a distal, general, and transdiagnostic individual risk factor (Ein-Dor et al., 2016). Its impact on grief may occur indirectly through unfinished business which acts as a proximal, loss-specific cognitive mechanism reflecting the individual’s ongoing cognitive appraisals of their relationship with the deceased.

### 4.3. Clinical Implications

The present study has several important clinical implications. First, although the deceased-specific attachment style did not directly predict long-term grief, its significant association with concurrent grief symptoms suggests that assessing the bereaved person’s attachment pattern toward the deceased remains important in clinical practice (Schenck et al., 2016). Attachment-informed grief therapy has also emphasized that the therapeutic focus should be guided by the client’s attachment style toward the deceased rather than by their global attachment tendencies (P. S. Kosminsky & Jordan, 2023). During therapy, the clinician can serve as a transitional attachment figure, helping bereaved individuals practice emotional regulation and flexible coping within a safe relational context (P. Kosminsky, 2022). Specifically, for bereaved individuals with high attachment anxiety, therapy may focus on reducing excessive psychological dependence on the deceased, promoting reality-oriented attention and self-care behaviors, and helping them reestablish balance between grief and daily life. For those with high attachment avoidance, interventions should gently foster awareness and acceptance of loss-related emotions and needs, encourage emotional expression and integration of the loss experience, and thus prevent long-term emotional numbing and relational detachment (Sekowski & Prigerson, 2022).

Second, unfinished business, representing cognitive and emotional content directly related to grief, serves as an important proximal risk factor and may hold potential as a more specific intervention target than attachment style. In clinical practice, directly addressing unfinished business may help alleviate concurrent grief more rapidly and effectively. Specifically, therapists can attend to clients’ verbal cues of unfinished business (e.g., “There are still things I never got to say,” “I still can’t forgive them,” “I wasn’t there when they needed me”). Previous research has shown that unfinished business can naturally foster continuing bonds with the deceased, and internalized bonds may help reduce the severity of grief (Huang et al., 2025). Therefore, clinicians can employ various techniques to help clients reconnect with the deceased, facilitate the expression of unfinished emotions, and promote cognitive integration and emotional relief (Holland et al., 2020). For example, letter writing, as an expressive writing approach, can provide a relatively safe structure for emotional contact with avoided material and subsequent integration; prior work suggests that writing a letter to the deceased may help individuals process unfinished business and aid in building continuing bonds (Larsen, 2024). Story retelling or narrative reconstruction can help clients recount their relationship with the deceased and the loss in a more coherent manner, identify key moments and core themes, and incorporate conflictual or complex emotions into a more balanced narrative while acknowledging attachment and longing (Mathew, 2023). Empty-chair work or imaginal dialogue, guided by the therapist, can facilitate a “conversation” with the deceased to express what remains unsaid, raise unresolved questions, and practice a more reparative response; careful pacing and appropriate grounding are essential (Gamoneda et al., 2025).

Clinicians can tailor interventions around unfinished-business work based on clients’ attachment-related coping styles. For clients with high attachment anxiety, interventions should focus more on reducing repetitive rumination, self-blame, and fostering self-compassion, while also helping them develop more realistic self-views and relationship appraisals. For clients with high attachment avoidance, clinicians may adopt a gradual, autonomy-supportive approach, selecting less threatening interventions that align with the client’s readiness (e.g., brief writing tasks or structured narration). Dialogue-based techniques can be used selectively and flexibly, depending on the client’s tolerance and therapeutic goals.

Clinicians should also tailor these techniques to align with cultural norms regarding grief expression and interpersonal interactions. In cultural contexts emphasizing emotional restraint and relational harmony (e.g., Chinese cultural contexts), clinicians may start with more indirect, structured approaches (e.g., writing-based exercises, narrative review) and gradually introduce more evocative dialogical techniques (e.g., empty-chair work, imaginal dialogue), with close attention to clients’ pace and tolerance (Hsu et al., 2009; Yick & Gupta, 2002). Collaboratively aligning the therapeutic frame, language, and pacing with clients’ values and cultural norms may enhance feasibility, acceptability, and cultural fit of the intervention.

In practice, tailoring may further draw on case-specific characteristics, including demographic and loss-related factors. Although subgroup or interaction analyses were beyond the scope of the present study, factors such as age, developmental stage, sex-linked emotional expression norms, cause of death (e.g., sudden/traumatic loss), and the relational context of the loss may help inform which unfinished-business themes to prioritize and how to pace intervention delivery (Holland et al., 2020; Huang et al., 2025). This case-by-case approach may guide both the selection of techniques and the level of emotional intensity introduced at each stage of therapy.

In summary, by assessing the bereaved individual’s attachment style toward the deceased and identifying as well as addressing unfinished business, clinicians can provide more targeted and timely psychological support, thereby facilitating adaptive grief recovery.

### 4.4. Limitations and Future Directions

The present study explored the relationships among attachment style, unfinished business, and grief. However, several limitations should be acknowledged. First, the representativeness of the sample is limited. Participants were primarily young adults from urban areas with relatively high educational levels. Therefore, caution is needed when generalizing these findings to populations such as rural residents, individuals with lower educational attainment, or older bereaved adults. Moreover, most participants reported the death of grandparents, whereas losses of core family members (e.g., parents, children, or spouses) were less common. This limitation prevented us from systematically examining whether the observed patterns differed across loss types. Future studies should recruit more diverse bereaved samples across key demographic characteristics (e.g., age, sex, education) and loss-related factors (e.g., cause of death). Such efforts would enhance generalizability of findings and provide an opportunity to test whether the attachment–unfinished business–grief associations vary across these characteristics. For example, moderation and moderated mediation analyses could help clarify for whom and under what conditions unfinished business is most clinically relevant.

Second, in terms of methodological design, although a longitudinal approach was adopted, the present study included only two time points with a relatively short interval (3 months). Prior research with three waves using the same 3-month interval suggests that while some associations were observed across adjacent waves, they were not consistently evident over more distal time points (Huang et al., 2025). This indicates that a two-wave, short-lag design may be inadequate for capturing the longer-term dynamics of grief, including delayed, non-linear, or stage-dependent change. Future research should incorporate more waves of data collection (three or more) and extend the follow-up period (e.g., longer intervals and/or multiple lag lengths) to better examine the reciprocal influences among attachment style, unfinished business, and grief. Building on these points, future studies may expand the model by exploring whether attachment-related processes vary depending on individual and contextual factors, such as social support or resilience (e.g., through moderation/moderated mediation), and by more precisely characterizing how unfinished business might differ across various stages of grief.

Despite these limitations, the current study is the first to examine the effects of deceased-specific attachment styles on grief from a longitudinal perspective. The results indicate that the effects of attachment styles may be more immediate than long-lasting. Furthermore, the study investigated the mediating role of unfinished business and found that deceased-specific attachment avoidance indirectly influenced grief through unfulfilled wishes. This finding extends our understanding of how attachment styles shape adaptation following loss and offers a new explanation for why previous studies have failed to demonstrate a prospective effect of deceased-specific attachment. Specifically, it suggests that the influence of attachment style on grief may occur indirectly through proximal, loss-specific cognitive processes, such as unfinished business. Finally, the current study underscores the potential clinical value of unfinished business as a target for intervention, providing empirical support for the development of more precise grief intervention strategies.

## 5. Conclusions

This two-wave longitudinal study clarifies the mechanisms linking deceased-specific attachment styles with grief. Although deceased-specific attachment anxiety and avoidance did not directly predict subsequent grief, deceased-specific attachment avoidance exerted an indirect effect through unfulfilled wishes, suggesting that unfinished business may play a meaningful role in grief adaptation. For palliative and bereavement care practice, these findings imply that interventions targeting unfinished business may offer a more focused and clinically efficient approach than broad attachment-based strategies, helping bereaved individuals adjust more adaptively after loss.

## Figures and Tables

**Table 1:** Sample characteristics for the final and attrition samples.

	N (%)/M ± SD		t/χ^2^	*p*
	Final Sample (N = 206)	Attrition Sample (N = 122)		
Demographic variables				
Age	25.07 ± 6.41	24.87 ± 5.58	−0.29	0.78
Gender				
Men	99 (48.06%)	60 (49.18%)	0.04	0.91
Women	107 (51.94%)	62 (50.82%)		
Educational level			7.29	0.06
Junior high school or below	5 (2.43%)	7 (5.74%)		
High school	23 (11.17%)	18 (14.75%)		
Undergraduate	164 (79.61%)	95 (77.87%)		
Postgraduate	14 (6.80%)	2 (1.64%)		
Loss-related variables				
Age of the deceased	67.97 ± 19.18	69.08 ± 18.22	0.52	0.61
Gender of the deceased				
Men	119 (57.77%)	69 (56.56%)	0.05	0.91
Women	87 (42.23%)	53 (43.44%)		
Time since loss (months)	16.19 ± 9.79	17.88 ± 10.69	1.45	0.15
Relationship to the deceased			7.35	0.27
Grandparents	148 (71.84%)	87 (71.31%)		
Parents	20 (9.71%)	12 (9.84%)		
Siblings	3 (1.46%)	5 (4.10%)		
Children	1 (0.49%)	1 (0.82%)		
Partners/Spouses	5 (2.43%)	2 (1.64%)		
Friends	13 (6.31%)	2 (1.64%)		
Other relatives	16 (7.77%)	13 (10.66%)		
Cause of death			4.58	0.19
Chronic illness	103 (50.00%)	50 (40.98%)		
Sudden illness	64 (31.07%)	52 (42.62%)		
Accident	37 (17.96%)	19 (15.57%)		
Suicide	2 (0.97%)	1 (0.82%)		

Note. In the relationship to the deceased category, other relatives include aunt, uncle, cousin, and great-grandparent.

**Table 2:** Correlations among deceased-specific attachment style, unfinished businesses, and grief (N = 206).

	M	SD	1	2	3	4	5	6	7
1. T1 deceased-specific attachment anxiety	2.17	1.53							
2. T1 deceased-specific attachment avoidance	2.22	0.99	0.25 ***						
3. T1 unfulfilled wishes	3.95	0.67	0.23 **	−0.29 ***					
4. T1 unresolved conflicts	2.83	0.92	0.41 ***	−0.06	0.44 ***				
5. T2 unfulfilled wishes	3.63	0.78	0.09	−0.30 ***	0.55 ***	0.22 **			
6. T2 unresolved conflicts	2.63	0.97	0.32 ***	−0.07	0.30 ***	0.61 ***	0.57 ***		
7. T1 grief	3.19	0.83	0.36 ***	−0.28 ***	0.56 ***	0.58 ***	0.48 ***	0.49 ***	
8. T2 grief	2.78	0.87	0.29 ***	−0.17 *	0.40 ***	0.47 ***	0.57 ***	0.70 ***	0.60 ***

Note. * *p* < 0.05, ** *p* < 0.01, *** *p* < 0.001.

## Data Availability

The original data presented in the study are openly available at https://osf.io/qy2tj/overview?view_only=acc8b046670f483d9ce8a768906b84ce (accessed on 9 January 2026).
